# Advances in Functional Rubber and Elastomer Composites

**DOI:** 10.3390/polym16121726

**Published:** 2024-06-17

**Authors:** Md Najib Alam

**Affiliations:** School of Mechanical Engineering, Yeungnam University, 280, Daehak-ro, Gyeongsan 38541, Republic of Korea; mdnajib.alam3@gmail.com

## 1. Introduction

Two crucial innovations—mastication and vulcanization—have revolutionized the use of rubber in our daily lives [1,2]. Initially, natural rubber was utilized to manufacture shoes, waterproof jackets, toys, and other items; however, the discovery of vulcanization in the mid-19th century has significantly transformed its applications. For example, solid rubber strips started to be used in wheels, greatly enhancing riding comfort in vehicles. With continuous advancements in science and manufacturing technologies, modern tires are now easier to produce, as well as being more durable, safer for driving, and more fuel-efficient.

At the beginning of the 20th century, the demand for rubber surged, but its supply was limited, prompting the search for new sources [3]. In 1909, a team led by Fritz Hofmann at Bayer laboratory successfully created the first synthetic rubber from isoprene monomers. In 1931, another synthetic rubber, neoprene, was synthesized, and a few years later, in 1935, a series of synthetic rubbers known as Buna rubbers were developed through the copolymerization of two monomers. Subsequently, many types of synthetic rubbers have been produced, featuring a wide range of chemical, thermal, and oil-resistant properties [3]. These special features enabled their use in applications such as oil fields and machinery.

Recently, elastomeric materials have found applications in advanced electronic devices; artificial intelligence; and robotics, medical, and health monitoring technologies [4,5,6]. For these applications, rubber or elastomers must possess special characteristics. For instance, elastomers can be made electrically or magnetically sensitive by incorporating electrically conductive or magnetic fillers [7,8,9]. These materials can then be used in smart sensing devices. Additionally, rubber composites with specific fillers exhibit excellent electromagnetic wave absorption properties. Some rubber composites can even function as transducers, converting mechanical energy into electrical power, which can be utilized for generating green energy [10,11].

Fillers are immensely important for making rubber composites for various applications. However, some fillers exhibit low performance abilities due to their surface characteristics, while others are expensive or pose human and environmental toxicity risks. In such cases, alternative or modified fillers can be beneficial [12,13]. Additionally, modifying rubber itself can enhance composite properties. By adopting these strategies, rubber composites have the ability to become more advanced, practical, cost-effective, and sustainable for our societies and industries.

## 2. Overview of Published Articles

Fasolt et al. [14] found that electrodes impact the breakdown voltage in dielectric elastomer actuators, which can be improved using stiffer, silicone rubber-based electrodes. Furthermore, Liu et al. [15] used hydrophobized, bio-based, microfibrillated cellulose as a reinforcing filler in silica/styrene butadiene rubber (SBR) tire tread compounds, observing a notable increase in mechanical properties compared to the sole use of silica/SBR compounds. Meanwhile, Wang et al. [16] enhanced the mechanical, dielectric, and hydrophobic properties of commonly used Methyl Vinyl Silicone Rubber by blending it with Fluorosilicone Rubber. Carbon black and silica remain dominant fillers in the rubber industry; Magaletti et al. [17] utilized modified carbon black with a bio-sourced Janus molecule to reinforce rubber, significantly reducing energy dissipation compared to conventional silica or carbon black-reinforced rubber compounds. Moreover, Song et al. [18] improved silica filler dispersion in rubber by using liquid styrene butadiene rubber end-functionalized with a silane coupling agent, resulting in significant improvements in rolling resistance, snow traction, and abrasion resistance compared to the traditionally used treated distillate aromatic extract (TDAE) oil as a filler dispersant. Slobodinyuk et al. [19] synthesized new shape memory polymers for self-healing coatings using oligomers with terminal epoxy groups from oligotetramethylene oxide dioles; they developed a high-yield synthesis method for oligoetherdiamines (94%), involving acrylic acid and aminoethylpiperazine, which enhanced the thermal and mechanical properties of urethane polymers, achieving over 95% shape fixity and over 94% shape recovery. Ahmed A. Bakhsh [20] explored the mechanical and thermal properties of polyolefin–hydroxyapatite nanocomposites using HDPE and LDPE matrices, finding significant enhancements with minor decreases at the 40% level of hydroxyapatite loading. Razzaq et al. [21] reported the 4D printing of electro-active, triple-shape composites made from polyester urethane (PEU), polylactic acid (PLA), and multiwall carbon nanotubes (MWCNTs). These composites, suitable for fused filament fabrication, demonstrated the triple-shape effect through resistive heating, offering potential applications in space, robotics, and actuation technologies. Additionally, Al-Mhyawi et al. [22] developed an adsorbent hydrogel using acrylic acid and orange peel via free radical polymerization to remove methylene blue (MB) from water; optimized and characterized using SEM and BET analysis, the hydrogel showed an 84% adsorption in 10 min and proved to be reusable for up to ten times, demonstrating an efficient and eco-friendly method for water treatment. Jung et al. [23] studied the effects of carbon black (CB) and silica fillers on H_2_ permeation in sulfur-crosslinked ethylene propylene diene monomer (EPDM) polymers. CB-blended EPDMs exhibited dual sorption, while neat and silica-blended EPDMs followed Henry’s law. CB-filled EPDMs reduced H_2_ diffusivity as a result of an increased tortuosity, suggesting its potential use as a sealant material for H_2_ refueling stations. Do et al. [24] investigated the mechanical responses of graded styrene–butadiene rubber (SBR) with varying crosslink densities compared to homogenously vulcanized SBR; graded SBR showed a good elasticity and a significant warpage after stress removal, indicating a prolonged shrinking stress on the high-crosslink surface, enhancing crack resistance and slow strain recovery. Alam et al. [25] explored using MgO as a co-activator to reduce conventional ZnO levels in rubber vulcanization. A 3:2 MgO:ZnO weight ratio significantly shortened the curing times and enhanced mechanical properties, providing a safer, high-performance alternative for industrial applications. Kumar et al. [26] developed stretchable magnetic composites using silicone rubber mixed with graphene nanoplatelets (GNPs) and electrolyte–iron particles (EIPs). These composites, cured at room temperature, exhibited enhanced mechanical and magnetic properties. GNPs provided high stiffness and stretchability, while hybrids of GNPs and EIPs showed an improved mechanical performance and magnetic sensitivity, which is ideal for soft robotics. Furthermore, Jung et al. [27] synthesized a series of bio-based thermoplastic polyurethanes (TPUs) using bio-based polyether polyol and 1,4-butanediol (BDO), with aromatic (4,4-methylene diphenyl diisocyanate: MDI) and aliphatic (bis(4-isocyanatocyclohexyl) methane: H_12_MDI) isocyanates. Various micro-phase structures were identified and matched with specific TPU samples, including H-BDO-2.0, M-BDO-2.0, H-BDO-2.5, and M-BDO-3.0. In another study, Kumar et al. [28] developed stretchable devices using silicone rubber composites with multi-wall carbon nanotubes (MWCNTs) and copper particles. The hybrid composites showed an optimal stiffness and stretchability, generating ~6 V with a cycle durability of over 0.4 million, making them suitable for flexible electronics and piezoelectric energy-harvesting applications. Yang et al. [29] examined the low-velocity impact response of sandwich plates with functionally graded carbon nanotube-reinforced composite (FG-CNTRC) face sheets and a Ti-6Al-4V auxetic honeycomb core. Using first-order shear deformation theory and Hamilton’s principle, they analyzed the impact response, considering various stacking sequences, CNT volume fractions, and impact conditions. In a review paper, Alamer et al. [30] discussed different carbon-based conductive materials on fabrics, notably carbon nanotubes and graphene, highlighting their superior properties and pivotal roles in electronic device applications across various fields

## 3. Summary and Future Outlook

High-level filler dispersion is crucial for optimizing the properties of rubber composites. In this Special Issue, numerous researchers have developed various techniques, both physical and chemical, to enhance filler dispersion. Despite these advancements, there remains a significant potential for improvement, particularly for functional elastomer composites that require specialized elastomers and fillers for specific applications. Researchers have created functional rubber composites, such as electroactive and magnetoactive rubber composites, which are promising for advanced engineering applications in robotics and sensing. Others have developed structural composites that are useful in separation, purification, and structural technologies. Curing studies of these functional composites are also essential for ensuring their reliability and to address environmental safety concerns, highlighting the need for future research in this area. The potential for advancements in the properties of functional rubber and elastomeric materials is boundless. Therefore, continued research in this special field is imperative for future progress.
